# Eye Care Utilization among Older Subjects with Visual Impairment in Northwest Ethiopia

**DOI:** 10.18502/jovr.v18i3.13779

**Published:** 2023-07-28

**Authors:** Aragaw Kegne Assaye, Melkamu Temeselew Tegegn, Gizachew Tilahun Belete

**Affiliations:** ^1^Department of Optometry, School of Medicine, College of Medicine and Health Sciences, University of Gondar, Gondar, Ethiopia; ^2^Optometry and Vision Science, School of Medicine, University of New South Wales, Sydney, Australia

**Keywords:** Eye Care Services, Ethiopia, Visual Impairments

## Abstract

**Purpose:**

To find out the level of eye care service utilization and its determinants among the elderly visually impaired populations while visiting ophthalmic outreach locations in North-Western Ethiopia, 2021.

**Methods:**

An ophthalmic outreach-based cross-sectional study was conducted on 852 visually impaired older people. Participants were selected by using a systematic random sampling method from January to July 2021. Data were collected by using an interviewer-administered questionnaire and an ocular examination. The collected data were entered into the Epi Info 7, and analyzed using SPSS 20. A binary logistic regression was fitted.

**Results:**

A total of 821 participants, with a response rate of 96.5%, were included in the study. The utilization of eye care services within the past two years prior to the study was 21.1% (95 % CI: 18.2–23.9). Having systemic disease (AOR = 3.2, 95% CI: 1.5–7.0), being a spectacle wearer (AOR = 4.5, 95% CI: 2.0–9.4), having visual impairment at distance (AOR = 2.9; 95% CI: 1.5–5.6), being blind (AOR = 2.9; 95% CI: 1.5–5.6), duration of visual impairment ≤ 1 year (AOR = 2.5; 95% CI: 1.3–4.9) were all significantly associated.

**Conclusion:**

In this study, utilization of eye care services was low. Being visually impaired at distance, being blind, recent onset of visual impairment, being a spectacle wearer, and having systemic disease were all related to the use of eye care services. The commonest barriers to utilization of eye care services were financial scarcity and long distances between eye care facilities.

##  INTRODUCTION 

At least 2.2 billion people have a visual impairment globally; of these, around 1 billion people have a visual impairment that could have been prevented or is still unaddressed.^[1]^ The calculated use of eye care services is uneven, and is determined by the availability, accessibility, affordability, and acceptability of services as well as the distribution of eye conditions and visual impairments.^[2,3,4,5]^ About 43.3 million people are blind worldwide, of whom 89% of people with blindness or visual impairment are found in developing countries such as Ethiopia.^[1,6,7,8]^

Eye care service utilization by the population is the use of eye care services that has been provided in the society with the aim of preventing and treating ocular conditions, enhancing ocular well-being or gaining information about an individual's eye health status and prognosis. Eye care service utilization is a broadly investigated issue with important concerns and suggestions on the health condition of an individual and the community at large.^[6,9]^ The percentages of eye care service utilization by people in different areas across the world for the period ranging between one and five years prior to when the investigation was conducted include the following: a study in China, where 71.9% of the participants had never been to a hospital for examinations,^[10]^; in Myanmar, among older individuals 87.4% sought eye care; in Pakistan, 45.3% had an eye exam in the past year;^[11]^ in South India, 35.5% had a history of eye examinations;^[5]^ in USA, among visually impaired participants, 52% had not seen an eye care provider within the last year;^[12]^; in California (USA), 36% had an eye care visit in the past 12 months;^[13]^ in South Africa, 25.2% had an eye examination between two and five years;^[3]^ in rural Nigeria, 68% never had eye examinations before;^[9,14]^ and in Ethiopia, 40 % of the participants' failure to use an eye care service was due to indirect costs,^[15]^ Another recent study in Ethiopia showed that the proportion of individuals who accessed eye care services within the last two years prior to the study was 23.8%.^[6]^

Researches suggested that people who lived in rural areas were characterized by: lower income, older ages, minor ethnicities, refugee populations, women, and inaccessible eye care service when needed.^[2]^

Being an urban resident, having a higher educational status, a higher family monthly income, a history of eye disease, being aware of regular eye checkups, wearing spectacles, and being visually impaired were all positively associated with seeking access to eye care services. However, being female and residing in rural communities were negatively associated with seeking access to eye care. The measured influence of age and having systemic diseases in seeking access to healthcare varied in different studies.^[6,7,8,16,17,18]^

Researches revealed that the barriers to utilizing eye care services in different regions or countries included the following factors – cost, trust, communication, clinic accessibility, transportation, distance of the eye care service provider, perception that eye care is not needed due to old age, having good vision in the other eye, the need for escort and social engagement or cultural belief.^[9,14][16][17][19][20][21][22][23]^

Studying the utilization of eye care services and its associated factors at an ophthalmic outreach site is important in determining the major hindering factors toward considering utilization of eye care services. However, there was no such study at the ophthalmic outreach site in Ethiopia. Therefore, the main objective of this study was to determine the level of utilization of eye care services provided within a community and their associated factors among the visually impaired older population who attended ophthalmic outreach sites in Northwest Ethiopia in 2021.

In addition, this study will give baseline information to healthcare planners and policy makers to take the required measures toward alleviating the barriers to utilizing eye care services.

##  METHODS 

### Study Design, Period, and Area

An ophthalmic outreach-based cross-sectional study was conducted in two districts (Chauhit and Tekledingay) of the central Gondar zone, Northwest Ethiopia among the visually impaired older population from January to July 2021. The University of Gondar tertiary eye care and training center is the only tertiary center in the central Gondar zone that provides comprehensive clinical and community eye health services. It also serves as a major referral center for 14 million people living in the Amhara region, Northwest Ethiopia. The departments of optometry and ophthalmology at the University of Gondar are providing continuous community eye care services through an outreach program in collaboration with the Light for the World organization and other non-governmental organizations. According to the University of Gondar tertiary eye care and training center report, about 293,927 people received eye care services in the outreach program between 2008 and 2019. These services included medical therapy, cataract surgery, trichiasis surgery, refraction with optical correction, and referral services.

### Study Population and Eligibility Criteria

An older population group, aged ≥ 40 years, attending the ophthalmic outreach sites during the study period was selected for the study. However, those who were unable to answer the questionnaire due to mental health or communication problems were excluded from the study.

### Sample Size Determination and Sampling Procedure

A sample size was determined using a single population proportion formula with the assumption of the expected proportion of utilization of eye care service being 23.8%,^[6]^ 95% confidence level, 3% desired precision, and 10% nonresponse rate. According to these parameters, the final calculated sample size was 852. The study participants were selected by applying a systematic random sampling technique from a registered logbook.

### Operational Definitions

#### Utilization of an eye care service

If an individual reported that he/she had visited an eye care service provider center for an eye checkup or examination at least once in two years prior to the date of the research, it was considered as utilization of an eye care service. If no visits were made to an eye care service provider within this specified time period, the eye care service was considered as not being utilized.^[6]^

#### Distance visual impairment (VI)

Presenting distance visual acuity of worse than 6/12 to no light perception (NLP) in the worse eye. It was subcategorized as mild VI (presenting visual acuity [PVA < 6/12 to ≤6/18], moderate VI [PVA < 6/18 to ≤6/60], severe VI [PVA< 6/60 to ≤3/60], and blindness [PVA < 3/60 to NLP]).^[24]^

#### Ocular injury

Self-reported history of any previous injury to the eye.^[24]^

#### Near visual impairment

Binocular presenting near vision of ≤6/12 (N6) with the best-corrected distance visual acuity of ≥6/12.^[25,26]^

#### History of ocular surgery

Participants who had any eye surgery prior to data collection.

#### Awareness of causes and treatments for visual impairment

This was determined by the responses of participants who answered “yes” to the following questions: “Have you heard about the causes of visual impairment?” and “Have you heard about the treatments for visual impairment?”, respectively.^[27]^

### Data Collection Tool and Procedure

Data were collected by conducting both face-to-face interviews using a pretested structured questionnaire and ocular examinations. The pretest was done in the Kola Diba district, Gondar Zone, which was outside of the study area. The questionnaire consisted of sociodemographic and economic data, behavioral factors, past ocular and medical history, and clinical characteristics of the study participants. The questions were adapted from previous literature^[6,13,14,15,17,19,24,28]^

Distance visual acuity was assessed in each eye using a Snellen acuity chart with tumbling E optotypes at 6 m in good illumination. Similarly, binocular near visual acuity was tested by using a reduced Snellen acuity chart at 40 cm. To assess the causes of visual impairment, an anterior segment examination was performed using a pen torch with a 2.5 × magnified loupe, and a posterior segment examination was performed using a dilated direct ophthalmoscope.

### Data Processing and Analysis

After checking completeness and consistency of the data, it was coded and entered into the EPI Info version 7 (TM Andrew, GD, USA), and then exported into the Statistical Package for Social Science (SPSS) version 20 (IBM Corp., Armonk, NY, USA) for analysis. Descriptive statistics such as frequency distribution and measure of central tendency were used to summarize the descriptive part of the study. A binary logistic regression was fitted to identify factors associated with the utilization of eye care services, and the strength of this association was expressed using an adjusted odds ratio with a 95% of confidence interval. The significant variables were selected by using an enter variable selection technique, and fitness of the model was checked using the Hosmer and Lemeshow's goodness of fit. Variables with a *P*-value of < 0.05 were considered statistically significant.

##  RESULTS 

### Sociodemographic Characteristics of the Study Participants 

The study population consisted of a total of 821 participants with a response rate of 96.5%, of whom 256 had history of eye examination before data collection. The median age of the participants with its interquartile range was 57 ± 23 (IQR) years. Out of all participants, 486 (59.2%) were male, 508 (61.9%) were rural residents, and 458 (55.8%) were unable to read and write [Table 1].

**Table 1:** Sociodemographic and economic characteristics of the study participants attending the ophthalmic outreach sites in the central Gondar zone, Northwest Ethiopian, 2021 (number of participants = 821).


**Variables**			**Time since the last eye examination **			***P*** **-value**
	***n***	**No exam.**	** ≤ 2 years (%) **	**3–5 years (%)**	** > 5 years (%)**	
All	821	565	173 (21.1)	60 (7.3)	23 (2.8)	
**Age (yr) **			< 0.001
40–49	254 (30.9)	205 (80.7)	33 (13.0)	10 (3.9)	6 (2.4)	
50–59	185 (22.6)	133 (71.9)	36 (19.5)	13 (7.0)	3 (1.6)	
60–69	162 (19.7)	103 (63.6)	39 (24.1)	14 (8.6)	6 (3.7)	
≥ 70	220 (26.8)	124 (56.4)	65 (29.5)	23 (10.5)	8 (3.6)	
**Sex**			0.25
Male	486 (59.2)	325 (66.9)	109 (22.4)	40 (8.2)	12 (2.5)	
Female	335 (40.8)	240 (71.6)	64 (19.1)	20 (6.0)	11 (3.3)	
**Residence**			0.87
Rural	508 (61.9)	352 (69.2)	108 (21.3)	37 (7.3)	11 (2.2)	
Urban	313 (38.1)	213 (68.1)	65 (20.8)	23 (7.3)	12 (3.8)	
**Religion**			0.32
Christian	759 (92.6)	519 (68.4)	163 (21.5)	58 (7.6)	19 (2.5)	
Muslim	62 (7.6)	46 (74.2)	10 (16.1)	2 (3.2)	4 (6.5)	
**Marital status (currently)**			0.05
Single	156 (19.0)	99 (63.5)	42 (26.9)	9 (5.8)	6 (3.8)	
Married	665 (81.0)	466 (70.0)	131 (19.7)	51 (7.7)	17 (2.6)	
**Educational status**			0.71
Unable to read and write	458 (55.8)	323 (70.5)	98 (21.5)	29 (6.3)	8 (1.7)	
Able to read and write	149 (18.1)	97 (65.1)	35 (23.5)	13 (8.7)	4 (2.7)	
Primary and secondary school	112 (13.6)	83 (74.1)	20 (17.8)	4 (3.6)	5 (4.5)	
College and above	102 (12.4)	62 (60.8)	20 (19.6)	14 (13.7)	6 (5.9)	
**Occupational status**			0.2
Government employee	111 (13.5)	71 (63.9)	24 (21.6)	12 (10.8)	4 (3.6)	
Merchant	54 (6.6)	39 (72.2)	7 (12.9)	3 (5.6)	5 (9.3)	
Farmer	362 (44.1)	252 (69.6)	77 (21.3)	30 (8.3)	3 (0.8)	
Housewife	245 (29.8)	175 (71.4)	49 (20.0)	12 (4.9)	9 (3.7)	
Others*	49 (6.0)	28 (57.1)	16 (32.7)	3 (6.1)	2 (4.1)	
**Monthly income (Ethiopia birrs`)**				0.67
≤ 1000	402 (49.0)	265 (65.9)	92 (22.9)	32 (8.0)	13 (3.2)	
1001–15000	169 (20.6)	126 (74.6)	33 (19.5)	8 (4.7)	2 (1.2)	
1501–2000	78 (9.5)	57 (73.1)	15 (19.2)	5 (6.4)	1 (1.3)	
≥ 2001	172 (20.9)	117 (68.0)	33 (19.2)	15 (8.7)	7 (4.1)	
**Health insurance**			0.04
Yes	288 (35.1)	190 (66.0)	72 (25.0)	19 (6.6)	7 (2.4)	
No	533 (64.9)	375 (70.4)	101 (18.9)	41 (7.7)	16 (3.0)	
**Had escort**			0.4
Yes	680 (82.8)	459 (67.5)	147 (21.6)	55 (8.1)	19 (2.8)	
No	141 (17.2)	106 (75.2)	26 (18.4)	5 (3.6)	4 (2.8)	
	***n***	**No exam.**	** ≤ 2 years (%) **	**3–5 years (%)**	** > 5 years (%)**	
**Living arrangement**			0.09
Living alone	85 (10.4)	57 (67.1)	24 (28.2)	1 (1.2)	3 (3.5)	
With family members	736 (89.6)	508 (69.0)	149 (20.3)	59 (8.0)	20 (2.7)	
**Family size**			0.04
≤ 5	700 (85.3)	491 (70.1)	139 (19.9)	50 (7.1)	20 (2.9)	
> 5	121 (14.7)	74 (61.1)	34 (28.1)	10 (8.3)	3 (2.5)	
	
	
n, sample size; yr, years; others* included daily worker, retired, monk and unemployed The vertical sums in the second column (labeled *n*) are calculated out of the total sample size (821); the percentages of *n* = 821 are reflected in the brackets, and the horizontal sums (had examinations [labeled time since last examinations] plus no examinations) equals 100 as the percentage stated in the brackets The *P*-value is an output value of the binary logistic regression model						

### Medical and Ocular History of the Study Participants 

Of the total study participants, 47 (5.7%), 110 (13.4%), and 46 (6.0%) had a history of systemic hypertension, ocular surgery and wearing of spectacles, respectively [Table 2].

**Table 2:** Medical and ocular history of study participants attending ophthalmic outreach sites in the central Gondar zone, Northwest Ethiopia, 2021 (number of participants = 821).


**Variables**			**Time since the last eye examination **			***P*** **-value**
	***n***	**No exam.**	** ≤ 2 years (%)**	**3–5 years (%)**	** > 5 years (%)**	
All	821	565	173 (21.1)	60 (7.3)	23 (2.8)	
Known diabetes mellitus			0.88
Yes	27 (3.3)	15 (55.6)	6 (22.2)	5 (18.5)	1 (3.7)	
No	794 (96.7)	550 (69.3)	167 (21.0)	55 (6.9)	22 (2.9)	
Known hypertension			0.74
Yes	47 (5.7)	28 (59.6)	9 (19.1)	7 (14.9)	3 (6.4)	
No	774 (94.3)	537 (69.4)	164 (21.2)	53 (6.8)	20 (2.6)	
Known other systemic diseases*			0.04
Yes	38 (4.6)	21 (55.3)	13 (34.2)	1 (2.6)	3 (7.9)	
No	783 (95.4)	544 (69.5)	160 (20.4)	59 (7.5)	20 (2.6)	
History of ocular trauma			0.57
Yes	40 (4.9)	27 (67.5)	7 (17.5)	3 (7.5)	3 (7.5)	
No	781 (95.1)	538 (68.8)	166 (21.3)	57 (7.3)	20 (2.6)	
History of ocular surgery			< 0.0001
Yes	110 (13.4)	5 (4.5)	66 (60.0)	28 (25.5)	11 (10.0)	
No	711 (86.6)	560 (78.8)	107 (15.0)	32 (4.5)	12 (1.7)	
Use of spectacle			0.02
Yes	49 (6.0)	16 (32.6)	17 (34.7)	11 (22.5)	5 (10.2)	
No	772 (94.0)	549 (71.1)	156 (20.2)	49 (6.4)	18 (2.3)	
Use of ocular self-medication			0.22
Yes	46 (5.4)	28 (60.9)	13 (28.3)	3 (6.5)	2 (4.3)	
No	775 (94.6)	537 (69.3)	160 (20.6)	57 (7.4)	21 (2.7)	
Use of traditional medication			0.98
Yes	24 (2.9)	16 (66.7)	5 (20.8)	0 (0.0)	3 (12.5)	
No	797 (97.1)	549 (68.9)	168 (21.1)	60 (7.5)	20 (2.5)	
	
	
Note: Other systemic diseases* include Tuberculosis, Human Immunodeficiency Virus, Arteritis, Asthma, Malaria The vertical sums in the second column are calculated out of the total sample size (821), and the horizontal sums (had examinations plus no examinations) equals 100 No exam means that the participants didn't have eye examinations The *P*-value is an output value of the binary logistic regression model						

### Clinical Characteristics of the Study Participants 

A total of 513 participants with distance visual impairment and 308 participants with near visual impairments were involved in the study. In the current study, only 33.6% of the participants with blindness and 27.1% participants with cataract utilized eye care services within the past two years [Table 3] prior to the study.

**Table 3:** Clinical characteristics of study participants who attended ophthalmic outreach sites in central Gondar zone, Northwest Ethiopia (number of participants = 821).


**Variables**			**Time since the last eye examination **			***P*** **-value**
	***n***	**No exam.**	** ≤ 2 years (%) **	**3–5 years (%)**	** > 5 years (%)**	
All	821	565	173 (21.1)	60 (7.3)	23 (2.8)	
Types of visual impairment (VI)			< 0.0001
Distance VI	513 (62.5)	319 (62.2)	135 (26.3)	46 (9.0)	13 (2.5)	
Near VI	308 (37.5)	244 (79.2)	38 (12.4)	16 (5.2)	10 (3.2)	
Level of distance VI in the worse eye (*n *= 513)			< 0.0001
Mild	60 (7.3)	40 (66.7)	15 (25.0)	5 (8.3)	0 (0.0)	
Moderate	167 (20.3)	110 (65.8)	36 (21.6)	16 (9.6)	5 (3.0)	
Severe	149 (18.1)	93 (62.4)	38 (25.5)	15 (10.1)	3 (2.0)	
Blind	137 (16.7)	76 (55.5)	46 (33.6)	10 (7.3)	5 (3.6)	
Cause of visual impairment in the worse eye (*n *= 513)			0.02
Cataract	291 (35.4)	181 (61.9)	79 (27.1)	25 (8.6)	7 (2.4)	
Refractive error	84 (10.2)	60 (71.4)	16 (19.1)	7 (8.3)	1 (1.2)	
Corneal opacity	45 (5.5)	25 (55.6)	11 (24.4)	7 (15.6)	2 (4.4)	
Glaucoma	64 (7.8)	44 (68.8)	15 (23.4)	4 (6.2)	1 (1.6)	
Other*	29 (3.5)	10 (34.5)	14 (48.3)	3 (10.3)	2 (6.9)	
Duration of visual impairment (yr)			0.02
≤ 1	229 (27.9)	172 (75.1)	48 (21.0)	6 (2.6)	3 (1.3)	
2–4	443 (54.0)	293 (66.1)	106 (23.9)	37 (8.4)	7 (1.6)	
> 4	149 (18.1)	100 (67.1)	19 (12.8)	17 (11.4)	13 (8.7)	
Aware about the cause of VI			< 0.0001
Yes	223 (27.2)	35 (15.7)	132 (59.2)	42 (18.8)	14 (6.3)	
No	598 (72.8)	530 (88.6)	41 (6.9)	18 (3.0)	9 (1.5)	
Aware about treatment of VI			< 0.0001
Yes	219 (26.7)	33 (15.1)	131 (59.8)	41 (18.7)	14 (6.4)	
No	602 (73.3)	532 (88.4)	42 (6.9)	19 (3.2)	9 (1.5)	
	
	
Note: Other*: diabetic retinopathy, age-related macular degeneration, advanced pterigyum VI, visual impairment; yr, years The vertical sums in the second column are calculated out of the total sample size (821), and the horizontal sums (had examinations plus no examinations) equals 100 No exam, means that the participants didn't have eye examinations The *P*-value is an output value of the binary logistic regression model						

### Utilization of Eye Care Services among Visually Impaired Older Population 

Of the 821 study participants, 256 (31.2%) had previous history of eye examination before data collection, of which 175 (68.4%) had conducted an eye examination at an ophthalmic care hospital, whereas 81 (31.6%) had undergone an eye examination at an outreach ophthalmic site.

This study revealed that the proportion of utilization of eye care services within the past two years was 21.1% (95% CI: 18.2–23.9).

### Barriers to Utilization of Eye Care Services and Need of Eye Care Services 

**Figure 1:**
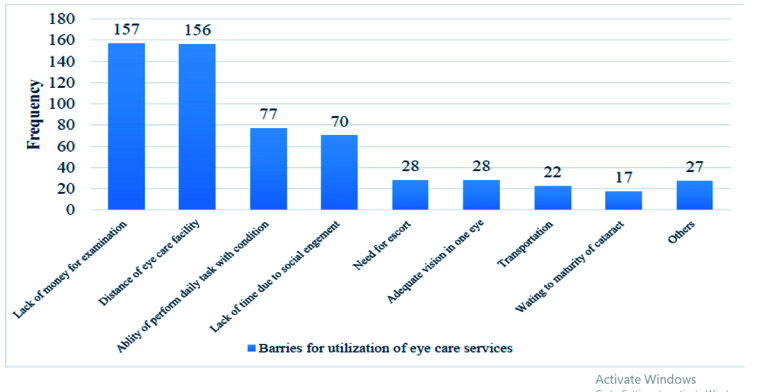
Self-reported barriers to utilize eye care services among visually impaired older population attending the ophthalmic outreach sites in the central Gondar zone, Northwest Ethiopia (*n* = 565).

The major barriers to utilizing eye care services reported by the participants included the following – a lack of money for eye examinations: 157 (27.8%), the distance of the eye care service provider: 156 (27.6%), and the ability to perform daily activities with the condition: 77(13.6%) [Figure 1].

In the present study, the most required eye care service in both female and male participants was cataract surgery [Table 4].

**Table 4:** Need of eye care service among study participants, Northwest Ethiopia (*n* = 821).


**Sex**	**Level of VI**	**Need of eye care service**						
	**Near correction (%)**	**Cataract Surgery (%)**	**Trichiasis surgery (%)**	**Refractive error correction (%)**	**Glaucoma follow-up (%)**	**Low vision rehabilitation (%)**	**Others (%)**
Male	Near (*n* = 182)	180 (98.9)	0 (0.0)	2 (1.1)	0 (0.0)	0 (0.0)	0 (0.0)	0 (0.0)
	Mild (*n* = 32)	0 (0.0)	12 (37.5)	1 (3.1)	7 (21.9)	3 (9.4)	1 (3.1)	8 (25.0)
	Moderate (*n* = 101)	0 (0.0)	50 (49.5)	3 (3.0)	27 (26.7)	12 (11.9)	3 (3.0)	9 (8.9)
	Severe (*n* = 88)	0 (0.0)	57 (64.7)	1 (1.1)	5 (5.7)	13 (14.8)	6 (6.8)	6 (6.8)
	Blind (*n* = 83)	0 (0.0)	48 (57.8)	0 (0.0)	3 (3.6)	17 (20.5)	8 (9.6)	4 (4.8)
	Total (*n* = 486)	180 (37.0)	167 (34.4)	7 (1.4)	42 (8.6)	45 (9.3)	18 (3.7)	27 (5.6)
Female	Near (*n* = 126)	123 (97.6)	0 (0.0)	3 (2.3)	0 (0.0)	0 (0.0)	0 (0.0)	0 (0.0)
	Mild (*n* = 28)	0 (0.0)	12 (42.8)	1 (3.6)	15 (53.6)	0 (0.0)	0 (0.0)	1 (3.6)
	Moderate (*n* = 66)	0 (0.0)	37 (56.1)	1 (1.5)	17 (25.8)	8 (12.1)	1 (1.5)	1 (1.5)
	Severe (*n* = 61)	0 (0.0)	41 (67.2)	0 (0.0)	6 (9.8)	7 (11.5)	2 (3.3)	4 (6.6)
	Blind (*n* = 54)	0 (0.0)	34 (63.0)	3 (5.6)	4 (7.4)	4 (7.4)	6 (11.1)	4 (7.4)
	Total (*n* = 335)	123 (36.7)	124 (37.0)	8 (2.4)	42 (12.5)	19 (5.7)	9 (2.7)	10 (3.0)
	
	
VI, visual impairment Mild VI (PVA < 6/12 – ≤ 6/18), Moderate VI (PVA < 6/18 – ≤ 6/60), Severe VI (PVA < 6/60 – ≤ 3/60), and Blindness (PVA < 3/60 – NLP)								

### Factors Associated with the Utilization of Eye Care Service among Visually Impaired Population 

A multivariable (binary) logistic regression analysis output showed that the presence of systemic disease, use of spectacles, history of ocular surgery, types of visual impairment, level of visual impairment at distance, and duration of visual impairment were significantly associated with the utilization of eye care services.

Participants who had systemic disease such as tuberculosis, HIV/ADS, and arthritis were 3.2 times more likely to have utilized an eye care service than those who did not have systemic disease (AOR = 3.2, 95% CI: 1.5–7.0). As compared to participants who did not use spectacles, participants who used spectacles were 4.5 times more likely to have utilized an eye care service (AOR = 4.5, 95% CI: 2.0–9.4). Similarly, participants who had a history of ocular surgery were 9.3 times more likely to utilize an eye care service than their counterparts (AOR = 9.3; 95% CI: 5.6–15.4).

Participants who had distance visual impairment (either bilateral or unilateral) were 2.9 times more likely to utilize an eye care service as compared to those who presented with near visual impairment (AOR = 2.9; 95% CI: 1.5–5.6). In addition, the odds of utilization of an eye care service were 2.0 times more likely for those participants with severe visual impairment (AOR = 2.0; 95% CI: 1.03–3.8) 2.9 times more likely for those with blindness (AOR = 2.9; 95% CI: 1.5–5.6) than the participants with normal vision at distance.

Participants who had a duration of visual impairment ≤ 1 year and 1–4 years were 2.5 times (AOR = 2.5; 95% CI: 1.3–4.9) and 2.7 times (AOR = 2.7; 95% CI: 1.5–5.0), respectively more likely to utilize an eye care service than participants who had a duration of visual impairment > 4 years [Table 5].

**Table 5:** Factors associated with utilization of eye care services within the past two years among the visually impaired population attending ophthalmic outreach sites in the central Gondar zone, Northwest Ethiopia (*n* = 821).


**Variables**	**Utilization of eye care service**		**COR (95%CI)**	**AOR (95%CI)**	***P*** **-value**
	**Yes**	**No**			
Age (yr)			0.67
40–49	33	221	1.00	1.00	
50–59	36	149	1.6 (1.0–2.7)	1.3 (0.7–2.2)	
60–69	39	123	2.1 (1.3–3.5)	1.0 (0.5–1.8)	
≥ 70	65	155	2.8 (1.8–4.5)	0.9 (0.5–1.6)	
Current marital status			0.09
Single	42	114	1.5 (1.01–2.2)	1.4 (0.9–2.4)	
Married	131	534	1.00	1.00	
Health insurance			0.14
Yes	72	216	1.4 (1.01–2.0)	1.3 (0.9–2.0)	
No	101	432	1.00	1.00	
Number of family			0.25
≤ 5	139	561	1.00	1.00	
> 5	34	87	1.6 (1.02–2.4)	1.4 (0.8–2.3)	
Known other systemic diseases			0.003
Yes	13	25	2.0 (1.01–4.0)	3.2 (1.5–7.0)	
No	160	623	1.00	1.00	
Use of spectacle			< 0.0001
Yes	17	32	2.1 (1.1–3.9)	4.5 (2.0–9.4)	
No	156	616	1.00	1.00	
History of ocular surgery			< 0.001
Yes	66	44	8.5 (5.5–13.1)	9.3 (5.6–15.4)	
No	107	604	1.00	1.00	
Types of visual impairment			0.002
Distance VI	135	378	2.5 (1.7–3.8)	2.9 (1.5–5.6)	
Near VI	38	270	1.00	1.00	
Level of visual impairment			0.03
Normal vision	38	270	1.00	1.00	
Mild VI	15	45	2.4 (1.2–4.7)	2.1 (0.9–4.6)	
Moderate VI	36	131	2.0 (1.2–3.2)	1.4 (0.8–2.7)	
Severe VI	38	111	2.4 (1.5–4.0)	2.0 (1.03–3.8)	
Blind	46	91	3.6 (2.2–5.9)	2.9 (1.5–5.6)	
Duration of visual impairment (yr)			0.004
≤ 1	48	181	1.8 (1.02–3.2)	2.5 (1.3–4.9)	
1–4	106	337	2.2 (1.3–3.7)	2.7 (1.5–5.0)	
> 4	19	130	1.00	1.00	
	
	
The *P*-value is an output value of the binary logistic regression model VI, visual impairment; yr, years					

##  DISCUSSION 

In this study, the proportion of the society using eye care services among the visually impaired older population was 21.1% (95% CI: 18.2–23.9). This finding was lower than the findings from studies conducted in Hawassa city, Ethiopia,^[6]^ Edo state, Nigeria,^[14]^ South Africa,^[3]^ rural South India,^[29]^ Pakistan,^[11]^ Myanmar (Asia),^[30]^ Iran,^[31]^ Peru,^[28]^ Oregon (USA),^[32]^ rural Alabama (USA),^[12]^ and California (USA).^[13]^ The discrepancy might be due to the variations of the sociodemographic characteristics of the study participants. For instance, the number of participants with a higher education level involved in the current study was small, and they were less educated than those in studies conducted in Hawassa, Ethiopia, and Nigeria. However, the level of education in this study did not show or did not have any significance with eye care service utilizations. In addition, participants of the study done in South Africa, USA, Peru, Iran, and Pakistan were older, had higher educational status, had health insurance and systemic diseases. Those conditions like being older in age could increase age-related eye disease, and having higher education level could also create awareness about the importance of accessing eye care services.

However, this measurement of the proportion of the society utilizing eye care services was higher than a study conducted in Ghana.^[33]^ The possible reason could be the study in Ghana was conducted at a food market where participants came to sell or to buy commodities. The increased number of rural participants who came for trade were more likely to be healthy because of their origin so their focus was on trade and not on seeking eye care services.

Participants who had systemic diseases such as tuberculosis, HIV/AIDS, and arthritis were 3.2 times more likely to have utilized an eye care service than those who did not have any systemic disease. This finding was in line with the studies conducted in Kenya,^[34]^ Pakistan,^[11]^ Peru,^[28]^ and Oregon, USA.^[32]^ These statistics justified that systemic diseases can aggravate the complications of vision-related disorders which leads to the prompting of seeking eye examinations. Furthermore, people with other diseases may have become more familiar with the healthcare system and are able to use the facilities more than those who have never visited a healthcare facility.

In this study, participants who had distance visual impairment were 2.9 times more likely to have utilized an eye care service as compared to participants who presented with near visual impairment; this revelation was supported by a study done in Oregon (USA).^[32]^ Distance vision is required for a great variety of daily tasks especially for farmers who do activities like ploughing, sowing, herding, and other work responsibilities. The majority of the participants in this study were farmers, whose activities were specifically at distance, as mentioned earlier. So, those individuals are much more affected by impairments related to distance vision than near vision.

In the present study, the likelihood of using an eye care service among the blind and severely visually impaired participants was 2.9 and 2.0 times, respectively, as compared to participants with normal vision at distance. This result was in agreement with the results from studies done in Ethiopia,^[15]^ South India,^[20]^ Tehran,^[18]^ and China.^[10]^

This study revealed that participants who used spectacles were 4. 5 times more likely to use eye care services than non-spectacle users. Similarly, participants who had a history of ocular surgery were 9.3 times more likely to have utilized an eye care service than their counterparts. The possible explanation for this association is that individuals who used spectacles and underwent ocular surgery may be required to visit an eye care center on a more regular basis for prognosis and checkups than those who did not use spectacles or did not experience ocular surgery.

In the present study, the duration of visual impairment was one of the significant factors associated with the utilization of an eye care service in which participants who had a duration of visual impairment of ≥ 4 years were 2.7 times more likely to have utilized an eye care service than those who had a duration of visual impairment of < 4 years. The possible justification is that immediate onset of visual impairment can affect the performance of daily activities and cause stress wondering whether the vision would be recovered or not. These conditions would lead to a visit to an eye care service provider center. On the other hand, an individual who lived with visual impairment for a long duration will lose their hopes for visual recovery which reduces the desire for utilization of eye care services.

In this study, the major barriers to utilizing eye care services reported by the participants included the following factors: lack of money and the distance of the eye care facility from the points of origin. This finding was in line with the studies done in the Gurage zone, Ethiopia,^[15]^ Edo state, Nigeria,^[14]^ India,^[5,29]^ China,^[10]^ and Florida, USA.^[12]^ This finding justifies that poverty and inaccessibility of eye care services have their own effects on the utilization of eye care services. In order to encourage the utilization of eye care services, adequate provision of free eye care services at community level through the outreach program is required.

In conclusion, in this study, the utilization of eye care services among the visually impaired population was low. History of ocular surgery, use of spectacles, level of visual impairment at distance, duration of visual impairment, and having systemic disease were significantly associated with the utilization of eye care services. The major barriers to utilizing eye care services reported by the participants were lack of money and the distance of the eye care facility.

We recommend that eye health stakeholder organizations develop strategies for improving community utilization of the eye care services through eye health education and the provision of free eye care services to low-income populations.

##  Ethical Considerations

This study was conducted with respect to the principle of the Declaration of Helsinki. Ethical approval was obtained from the University of Gondar, College of Medicine and Health Sciences, School of Medicine, Ethical Review Committee. After detailed explanation of the purpose of the study, written informed consent was obtained from all the participants. Participants were informed about their right to withdraw from the study at any time during the interview and examination. The selected study participants were not receiving any incentive because of their participation in the study. The study participants’ confidentiality was maintained by removing personal identifiers from the data collection tools and locking the data with a password.

##  Financial Support and Sponsorship

None.

##  Conflicts of Interest 

None.
